# Toward human-centred resilience quantification: The role of eco-affective state variables

**DOI:** 10.1371/journal.pmen.0000700

**Published:** 2026-09-15

**Authors:** Lucas Murrins Marques

**Affiliations:** 1 Mental Health Department, Santa Casa de São Paulo School of Medical Sciences, São Paulo, Brazil; 2 Eco-Wellbeing & Affective Health Laboratory (E.W.A.H. Lab), São Paulo, Brazil; PLOS: Public Library of Science, UNITED KINGDOM OF GREAT BRITAIN AND NORTHERN IRELAND

## Abstract

Resilience has become one of the most influential ideas in how societies plan for climate change and environmental risk, guiding decisions in disaster preparedness, urban planning, and sustainability policy. Yet most tools used to measure resilience focus on physical infrastructure, institutional capacity, and socio-economic indicators, and pay comparatively little attention to how people actually experience environmental change as it unfolds. This gap matters, because how communities feel about a changing environment, whether they feel distressed, disconnected, or motivated to act, shapes whether adaptation efforts can be sustained over time. This Essay argues that resilience quantification is incomplete without accounting for these lived, emotional responses, here termed eco-affective state variables: patterned, population-level affective responses that emerge from sustained human-environment interactions. I focus in particular on two such states, solastalgia (distress caused by unwelcome change to one's home environment) and soliphilia (an affective orientation of care and commitment toward one's environment), because together they capture both the psychological cost of environmental change and the affective resource that allows people to keep engaging with it. I propose that these states can be tracked as complementary indicators, moderating factors, and early-warning signals within existing resilience frameworks, without replacing conventional metrics or treating environmental distress as a clinical problem. Incorporating eco-affective states into resilience metrics can help identify communities under strain before this strain becomes visible in conventional indicators, with direct implications for urban planning, climate adaptation, and environmental public health.

## Introduction

Mental health is increasingly recognized as a critical dimension of global environmental change. Authoritative syntheses now conclude with high confidence that climate change is already adversely affecting mental health and wellbeing worldwide, influencing not only clinical outcomes but also coping, functioning, and social stability [1–4]. Despite this, mental health continues to be positioned primarily as an outcome of environmental disruption, rather than as a system-relevant signal of emerging vulnerability and adaptive capacity.

Before proceeding, it is useful to state explicitly which sense of resilience this Essay addresses, since the term is used differently across disciplines. In engineering, resilience typically denotes the capacity of a system to return to a prior state of equilibrium following disturbance; in ecology, the capacity of a system to absorb disturbance while retaining its basic structure and function; in psychology, an individual capacity to adapt positively to adversity; and in social-ecological systems research, the capacity of a coupled human-environment system to persist, adapt, and transform under changing conditions [5–7]. This Essay is concerned primarily with the latter sense, social-ecological and community resilience as applied within environmental governance, disaster risk reduction, and climate adaptation policy. The eco-affective framework developed here is not offered as a replacement for engineering or psychological conceptions of resilience, but as a way of bringing population-level human experience into the analytical boundaries of social-ecological resilience quantification specifically.

It is also useful to specify what is meant by environmental stressors in this context, since the concept spans several distinct temporal profiles. Acute stressors include discrete, time-bounded events such as heatwaves, floods, and wildfires; chronic stressors include persistent, cumulative exposures such as air pollution, biodiversity loss, and water scarcity; and slow-onset changes include gradual, often irreversible processes such as sea-level rise and land degradation [1]. These exposure types can generate distinct eco-affective signatures, discussed further below, and resilience frameworks that treat environmental stress as a single undifferentiated category risk obscuring meaningful differences in how populations experience and respond to them.

In parallel, resilience has become a central organizing concept for governing complex socio-environmental systems under uncertainty. Across climate adaptation, disaster risk reduction, and sustainability transitions, resilience is increasingly operationalized through indicators, composite indices, and benchmarking frameworks designed to support decision-making and accountability [1,6,7]. While these approaches have advanced the measurement of exposure, infrastructure performance, and institutional capacity, they continue to face a persistent limitation: they are often better at describing system performance under disruption than at explaining why similarly exposed systems diverge in their trajectories of adaptation and transformation [8].

A key reason for this gap lies in how human dynamics are represented. In most resilience frameworks, populations are treated as static carriers of vulnerability or capacity, typically operationalized through sociodemographic indicators such as income, education, or access to services. Although these variables are important, they fail to capture how environmental change is dynamically perceived, experienced, and translated into behavior, collective action, or disengagement. This omission is consequential, as environmental stressors propagate through psychological, social, and institutional pathways that shape the viability of adaptation processes over time [1,2].

At the same time, a rapidly growing body of research has begun to characterize affective responses to environmental change, including eco-anxiety, ecological grief, and solastalgia. This literature has evolved from conceptual descriptions to more integrative frameworks that clarify mechanisms, measurement approaches, and population-level relevance [8–10]. Importantly, these affective responses are not merely individual reactions; they can influence attention, risk perception, motivation, and engagement with environmental issues, thereby shaping collective trajectories of response and adaptation.

Despite these advances, resilience science and climate-related mental health research have largely evolved in parallel. As a result, most resilience metrics continue to quantify systems in terms of physical exposure, infrastructure robustness, and governance capacity, while overlooking the affective system states through which environmental change becomes socially actionable or socially destabilizing. This creates a critical blind spot. Systems may appear resilient according to conventional indicators while simultaneously undergoing erosion in human endurance, trust, and willingness to sustain adaptive responses.

This Essay advances a focused claim: mental health should be understood not only as an outcome of environmental change, but as a system-relevant signal within human-environment systems. Building on this premise, I introduce the concept of eco-affective state variables, defined as time-varying, population-level affective patterns that emerge from sustained environmental exposure and that shape, and are shaped by, adaptive capacity. Integrating such variables into resilience quantification does not replace existing metrics; rather, it strengthens their explanatory and predictive power by incorporating early signals of adaptive strain, social fragility, and the conditions under which environmental transitions become either viable or unstable.

### Why resilience quantification remains incomplete

Over the past two decades, resilience quantification has evolved from a largely conceptual ambition into a set of formalized measurement strategies. Current approaches rely on physical and infrastructural indicators, composite indices integrating social and environmental variables, and recovery-oriented models that track system performance under disruption [5–7]. These developments have been essential for translating resilience into an operational objective, enabling benchmarking, monitoring, and integration into policy and governance processes.

From a decision-making perspective, such approaches are appealing because they are standardizable. Indicator systems allow institutions to compare performance, allocate resources, and justify interventions using metrics that appear transparent and evidence-based [7]. However, this standardization introduces a structural limitation: resilience metrics tend to privilege what is stable, comparable, and readily quantifiable, often at the expense of processes that are dynamic, experiential, and unevenly distributed across populations [5].

As a result, resilience is frequently operationalized as a property that systems possess, rather than as a condition continuously negotiated through human-environment interactions. Within most frameworks, populations are represented through relatively static indicators, such as income, education, or institutional access, capturing vulnerability and capacity in aggregate terms. While these measures are informative, they fail to capture how environmental change is perceived, experienced, and translated into behavior over time. This limitation is not trivial. Environmental stressors propagate through psychological, social, and institutional pathways that directly influence whether adaptation is sustained, whether recovery remains viable, and whether resilience trajectories stabilize or deteriorate [1,2]. Yet these pathways remain only weakly represented in dominant resilience metrics.

A growing body of evidence indicates that environmental change is already adversely affecting mental health and wellbeing at population scale, with implications for coping, risk perception, and social functioning [1,3,4]. Despite this, affective dynamics are rarely incorporated into resilience quantification. This creates a critical mismatch: resilience frameworks aim to capture system adaptability under stress while excluding the very human processes through which stress becomes socially consequential.

The consequences are both conceptual and practical. First, current metrics are limited in their ability to detect early warning signals. Affective strain, loss of control, and declining place attachment often precede observable failures in infrastructure or governance, yet remain invisible to indicator systems focused on physical assets or post-event recovery [9]. Second, resilience frameworks struggle to anticipate social tipping points, including erosion of trust, disengagement from adaptation policies, and resistance to governance interventions, dynamics that can destabilize systems despite strong technical capacity [1].

Finally, resilience strategies that neglect human experience face challenges in sustaining long-term adaptation. Resilience is not a one-off achievement but an ongoing process that depends on public engagement, emotional endurance, and perceived legitimacy. When these conditions deteriorate, resilience may erode even in systems that perform well according to conventional indicators. The incompleteness of current resilience quantification, therefore, is not merely theoretical, it directly constrains the explanatory power, predictive value, and practical relevance of resilience metrics in real-world environmental governance.

### Eco-affective states as system variables

Eco-affective states as system variables are typically treated within dominant resilience and environmental frameworks as individual-level outcomes, secondary responses that follow exposure to environmental stressors. While appropriate for clinical or psychological analysis, this framing becomes insufficient when resilience is understood as an emergent property of coupled human-environment systems.

Recent advances across environmental psychology and sustainability science suggest a different interpretation: affective responses to environmental change are not random or purely individual, but patterned, socially distributed, and temporally dynamic [8,9]. Under sustained environmental stress, emotional responses tend to cluster within communities, co-vary with place-based exposures, and propagate through social networks and shared narratives. In this sense, emotions function as collective signals of how environmental change is being perceived and internalized at the population level. From a systems perspective, these affective patterns can be understood as emergent properties arising from repeated interactions between environmental conditions and social structures. They reflect not only exposure to environmental change, but also how that change is interpreted, negotiated, and embedded within cultural and institutional contexts [1]. Crucially, these dynamics influence feedback loops central to resilience, including trust in institutions, willingness to engage in adaptive behaviors, and the legitimacy of long-term environmental policies. Building on this perspective, I propose the concept of eco-affective state variables as a means of integrating affective dynamics into resilience quantification. Eco-affective state variables are defined as time-varying, population-level affective patterns that emerge from sustained human-environment interactions and that shape, and are shaped by, adaptive capacity. This definition is intentionally conceptual, specifying the role of these variables within system dynamics rather than prescribing specific measurement techniques.

Three features distinguish eco-affective state variables from conventional indicators. First, they are relational, reflecting shared appraisals and collective meaning-making processes rather than isolated individual experiences. Second, they are dynamic, capable of shifting over relatively short timescales in response to environmental signals or governance changes, often more rapidly than structural socio-economic indicators. Third, they are functionally consequential: eco-affective states influence behaviors and institutional trajectories that directly affect resilience, including cooperation, compliance, disengagement, and contestation [1,6]. Importantly, integrating eco-affective variables does not replace existing resilience metrics. Instead, it complements them by capturing how environmental change is experienced and internalized at the population level. This additional layer provides insight into the conditions under which technically sound adaptation strategies may fail due to declining social endurance or legitimacy. Among the range of affective responses associated with environmental change, two are particularly relevant for resilience analysis: solastalgia and soliphilia.

The affective landscape of environmental change extends well beyond these two constructs. Related states, including eco-anxiety, ecological grief, climate worry, and anticipatory loss, capture overlapping but distinct dimensions of how people respond emotionally to a changing environment, and recent taxonomic work has catalogued a strikingly wide vocabulary through which such responses are expressed [9]. Solastalgia and soliphilia are given particular emphasis here, rather than this broader landscape, for three reasons. First, together they form a conceptual axis rather than a single construct: solastalgia is diagnostic, indexing deteriorating environmental belonging, while soliphilia is enabling, indexing the affective capacity that sustains stewardship under conditions of loss; jointly, they capture both strain and resource within a single, minimal framework in a way that either construct alone, or a longer inventory of related constructs, would not. I develop the theoretical basis for this pairing, and its behavioral consequences for stewardship trajectories, more fully elsewhere [10]. Second, solastalgia and soliphilia are place-anchored, which aligns them with the spatial, place-based logic of resilience quantification, whereas constructs such as generalized eco-anxiety are less consistently tied to a specific locale. Third, solastalgia in particular already has an established, if fragmented, measurement tradition (see below), providing a more immediate pathway to operationalization than less standardized constructs. This is a deliberate scoping decision rather than a claim that other eco-affective constructs are less relevant to resilience; extending this framework to the broader affective landscape, including eco-anxiety and ecological grief, is an important direction for future work.

Solastalgia refers to distress associated with perceived environmental degradation while remaining in place. Within a resilience framework, it can be interpreted as an early signal of perceived environmental decline, reflecting a mismatch between environmental conditions and expectations of continuity, safety, or identity [9]. At the population level, increasing solastalgia may indicate adaptive strain, a condition in which cumulative emotional burden begins to erode coping capacity and willingness to sustain adaptive efforts. In contrast, soliphilia captures affective orientations characterized by care, attachment, and commitment to environmental protection. It can be understood as an affective resource that supports sustained engagement, collective action, and long-term stewardship under conditions of uncertainty. High levels of soliphilia may buffer the effects of environmental stress by reinforcing social cohesion and adaptive motivation [1,10].

The distribution of these affective states across populations is not random, and its non-randomness is directly relevant to resilience quantification. Solastalgia is disproportionately concentrated in communities that are most ecologically embedded and most environmentally exposed, including Indigenous communities, rural communities affected by agricultural intensification or extractive industry, and coastal or forest-edge communities most directly exposed to climate-driven ecological change [11,12]. These are frequently the same communities in which the institutional and material resources needed to build soliphilia as collective infrastructure, including stable governance, cultural continuity, and access to intact ecosystems, are least available [10]. A resilience framework that samples eco-affective indicators only in urban or well-resourced settings will therefore systematically underrepresent the populations for whom these signals are most informative.

Taken together, these states illustrate how eco-affective variables can function as system-relevant signals without requiring clinical diagnosis or normative assumptions. They capture how resilience is experienced and negotiated at the human level, providing insight into whether adaptation trajectories are likely to stabilize or deteriorate over time.

### Translating eco-affective states into resilience quantification

For eco-affective state variables to meaningfully contribute to resilience quantification, they must be measurable in ways that are rigorous, scalable, and compatible with existing assessment frameworks (see Table 1). Importantly, this does not require entirely new methodological paradigms. Rather, it involves leveraging established measurement approaches from public health, social science, and digital epidemiology, while reinterpreting affective patterns as system-relevant signals rather than isolated psychological outcomes [2].

**Table 1:** Conceptual positioning of eco-affective state variables within resilience quantification frameworks.

Dimension	Conventional resilience metrics	Eco-affective state variables
**Ontological status**	Structural and functional properties of systems	Emergent properties of coupled human–environment systems
**Primary analytical focus**	Infrastructure, institutions, and system performance	Population-level human experience of environmental change
**Conceptual role**	Descriptive and evaluative (how systems perform and recover)	Interpretive and anticipatory (how systems are perceived, strained, and sustained)
**Temporal sensitivity**	Low to moderate; changes often detected post-disruption	High; responsive to early and cumulative environmental stress
**Dynamic behavior**	Relatively stable or slowly varying indicators	Time-varying patterns sensitive to environmental and governance signals
**Scale of observation**	Asset, institutional, or system level	Distributed population-level patterns anchored in place
**Human representation**	Stakeholders, beneficiaries, or vulnerable groups	Dynamic components of system state
**Relationship to exposure**	Indirect, mediated through physical or socio-economic indicators	Directly linked to perceived and lived environmental conditions
**Function in resilience analysis**	Performance assessment and recovery benchmarking	Early warning of adaptive strain and endurance capacity
**Explanatory power**	Explains system robustness and recovery capacity	Explains divergence in resilience trajectories under similar conditions
**Role in benchmarking**	Core indicators for comparison across systems	Complementary and moderating variables enhancing interpretability
**Decision-making relevance**	Retrospective and diagnostic	Prospective and anticipatory
**Policy implications**	Guides investment and structural interventions	Informs timing, pacing, and social sustainability of interventions
**Risk if misapplied**	Overconfidence in technical capacity	Misinterpretation without contextual grounding
**Relationship to other metrics**	Foundational and necessary	Complementary, non-substitutive

Note: Eco-affective state variables are presented as complementary analytical components intended to enhance, not replace, conventional resilience metrics.

Validated survey instruments remain a foundational pathway. Higginbotham and colleagues demonstrated the feasibility of assessing solastalgia at population scale using the Environmental Distress Scale in a mining-affected Australian community, an early and influential example of translating population-level eco-affective assessment into a field-deployable instrument [13]. In recent years, measures of climate- and environment-related affect, including eco-anxiety and related constructs, have undergone increasing psychometric development and cross-cultural validation [8]. A recently proposed standardized, modular instrument battery organizes solastalgia, eco-anxiety, ecological grief, and related constructs into a single assessment protocol explicitly designed for population-level and surveillance use, addressing the fragmentation that has historically limited cross-study comparison in this literature [14]. When deployed longitudinally at population scale, these instruments can capture temporal shifts in perceived environmental strain and emotional endurance that remain invisible to static socio-economic indicators. Their relevance for resilience lies not in individual diagnosis, but in detecting aggregate patterns across time and place. Digital data streams provide a complementary layer of observation. Language-based indicators derived from social media and digital communication platforms can reveal shifts in collective emotional tone and perceived threat in near real time [15]. Similarly, emerging work suggests that behavioral and physiological signals may serve as proxies of stress-related affective states when analyzed with appropriate ethical safeguards [15]. Behavioral indicators, such as mobility patterns or engagement with environmental initiatives, can further contextualize these affective signals within broader adaptive processes [2].

The interpretive value of these data increases substantially when integrated with environmental exposure. Advances in geographic information systems and spatial epidemiology enable affective indicators to be aligned with environmental conditions such as heat, pollution, or ecological degradation [4,16]. A spatial framework explicitly designed for this kind of integration has recently been proposed for urban systems, linking environmental exposure layers, landscape configuration, and person-place interaction to affective indicators at neighborhood scale [17]. This integration allows eco-affective states to be interpreted as place-based responses rather than abstract sentiments, strengthening their relevance for resilience analysis.

Once measured, eco-affective variables can be incorporated into resilience frameworks in three complementary ways. First, they can function as an additional layer of indicators, enriching existing resilience indices without displacing core physical or institutional metrics. In this role, they enhance explanatory depth by capturing how environmental conditions are experienced and internalized, helping explain why similar levels of exposure or investment may lead to divergent outcomes. Second, eco-affective variables can operate as moderating factors within resilience models. The effectiveness of infrastructure or governance interventions may depend on prevailing affective conditions, such as trust, perceived control, or emotional fatigue. Systems with comparable structural capacity may follow different trajectories if one experiences rising affective strain while the other maintains strong social engagement. Third, eco-affective indicators can act as early signals of emerging vulnerability. Affective strain often precedes observable breakdowns in infrastructure or institutional performance, allowing eco-affective metrics to identify latent risks in systems that otherwise appear functional [1]. This anticipatory capacity is particularly relevant for governance, where delays in detection can translate into costly or irreversible system failures. These roles are especially important in comparative assessments. Benchmarking exercises often assume that similar scores reflect comparable resilience. Incorporating eco-affective variables challenges this assumption by revealing differences in system endurance and adaptive viability that are not captured by conventional metrics alone. At the policy level, eco-affective indicators support a shift from reactive resilience toward anticipatory governance. By detecting early signals of strain, such as declining place attachment or increasing emotional burden, decision-makers can adjust communication strategies, pacing of interventions, and participatory processes before disengagement or resistance emerges [6].

How eco-affective variables interact, concretely, with existing resilience quantification frameworks warrants explicit treatment, since the value of the proposed approach depends on interfacing with, rather than displacing, established indicator systems. In place-based resilience frameworks such as the Disaster Resilience of Place model or composite disaster-resilience indices [7], human dimensions are conventionally operationalized through static socio-demographic proxies for vulnerability and capacity, such as income, education, and age structure. Eco-affective variables interact with these existing components along the same three roles described above: as an additional indicator layer computed alongside, and reported side-by-side with, existing socio-demographic sub-indices rather than folded into a single composite score, preserving the interpretability of both; as a moderator that is tested statistically for interaction effects with existing structural or institutional resilience sub-indices, clarifying when high infrastructural capacity does, and does not, translate into sustained adaptive behavior; and as a leading indicator monitored on a separate, higher-frequency track than the typically slow-changing socio-demographic indicators that dominate current frameworks, so that shifts in eco-affective state can trigger review of a system's resilience classification between scheduled full assessments. The worked example below illustrates this interaction concretely.

A brief worked example illustrates how this integration could operate in practice. Consider a Brazilian coastal municipality experiencing recurrent flooding and progressive mangrove loss. A conventional resilience assessment of this setting would characterize the affected neighborhoods through indicators such as household income, housing quality, and drainage infrastructure capacity [5,7], and might, on this basis, classify the area as moderately resilient given recent infrastructure investment. Under the framework proposed here, this assessment would be complemented by three eco-affective signals. First, a population-level solastalgia score, aggregated at the neighborhood level using a standardized instrument battery [13,14], functioning as an additional indicator reported alongside infrastructural metrics rather than merged into them. Second, a soliphilia measure collected among residents engaged in participatory mangrove restoration, functioning as a moderating variable that helps explain whether restoration investment is likely to translate into durable local stewardship or into externally enforced, and therefore fragile, compliance. Third, longitudinal, geographically referenced monitoring of the solastalgia-soliphilia ratio across flood-exposure zones, using the kind of spatial integration described above [17], functioning as an early-warning indicator reviewed on a monthly or quarterly cycle rather than at the multi-year intervals typical of infrastructural resilience assessment. In this illustration, eco-affective indicators might reveal declining soliphilia and rising solastalgia among long-term residents even where infrastructural indicators suggest adequate resilience, a divergence signalling that the pace or design of current adaptation measures is exceeding the community's affective and social capacity to sustain engagement, despite apparently intact physical resilience. Detecting this divergence early, rather than only once it manifests as declining stewardship or participation, is the central practical contribution eco-affective quantification is intended to make.

Importantly, this approach does not medicalize governance or reduce resilience to psychological outcomes. The focus remains on population-level patterns and system dynamics. When used responsibly, eco-affective indicators function as contextual signals that enhance the ability of resilience frameworks to capture how systems are actually experienced and sustained over time.

### Implications for environmental decision-making

Integrating eco-affective state variables into resilience frameworks has direct implications for how environmental policies are designed, implemented, and sustained. Current approaches in urban planning and environmental governance prioritize infrastructural robustness, land-use regulation, and service continuity, dimensions that are essential but insufficient for understanding how populations experience ongoing environmental change [5,6].

Eco-affective indicators provide an additional layer of insight by capturing how environmental transformation is perceived, internalized, and negotiated at the population level. This information is critical for anticipating public receptivity to long-term adaptation strategies. Policies that are technically sound may nevertheless fail if they exceed the limits of emotional endurance or are experienced as disruptive or alienating [1,11]. In climate adaptation, where interventions unfold over extended time horizons, eco-affective variables can help align technical feasibility with social sustainability. Tracking shifts in affective states allows policymakers to identify when adaptation strategies are approaching limits of acceptance, enabling adjustments in pacing, communication, or participatory design before disengagement or resistance emerges.

Environmental public health similarly benefits from this perspective. Traditional indicators focus on exposure and disease outcomes, which are essential but often reactive. Eco-affective signals provide earlier insight into pressures on wellbeing and social functioning, supporting preventive approaches that intervene before clinical thresholds are reached [2,3]. Beyond policy design, eco-affective variables are particularly relevant for governance and ongoing system management. Integrating eco-affective indicators expands monitoring to include human-system interaction, enabling detection of shifts in trust, engagement, and perceived legitimacy that may compromise long-term resilience. This supports more adaptive forms of governance, where strategies are iteratively refined based on both material and experiential feedback [1].

Importantly, eco-affective variables do not replace conventional metrics. They complement them by capturing whether systems remain socially viable and sustainable over time. When interpreted alongside environmental and institutional data, eco-affective signals provide a more complete picture of resilience, one that reflects not only whether systems function, but whether they remain livable and acceptable from the perspective of those who inhabit them.

### Methodological and ethical considerations

Integrating eco-affective state variables into resilience quantification raises important methodological and ethical challenges, particularly related to context sensitivity, comparability, and data governance. Affective responses to environmental change are inherently context-dependent, shaped by cultural meanings and place-based relationships with environments and institutions [8,9].

This context sensitivity introduces risks of misinterpretation if affective data are aggregated without adequate grounding. Eco-affective indicators should therefore be interpreted relative to place-specific baselines and complemented with contextual information. This is especially important for communities with disrupted place histories, including displaced persons and communities experiencing rapid land-use change, and for constructs such as solastalgia and ecological grief whose referents vary markedly across urban, rural, agricultural, coastal, and Indigenous contexts and therefore require explicit cultural adaptation rather than direct translation [14]. Spatial integration provides a critical anchor, aligning affective data with environmental exposures and socio-political conditions [4,16]. Beyond methodological considerations, the use of eco-affective data raises ethical concerns. Data derived from digital traces or behavioral patterns may blur the boundary between public health monitoring and intrusive surveillance if not governed transparently [2,15].

A critical distinction lies in how such data are used. When eco-affective indicators support early intervention and participatory governance, they function as tools of care. When used to enforce compliance or suppress dissent, they risk undermining trust and exacerbating vulnerability. Robust data governance, ensuring transparency, privacy protection, and public oversight, is therefore essential. Addressing these considerations is central to ensuring that eco-affective variables enhance, rather than compromise, the scientific and societal value of resilience quantification.

### A roadmap for integrating eco-affective variables into resilience science

In the short term, progress can be achieved by embedding eco-affective variables within existing resilience frameworks. Many current indices already incorporate social dimensions; adding eco-affective indicators as supplementary layers enables feasibility testing without disrupting established workflows. Pilot studies are a critical next step. Place-based implementations can assess the sensitivity and policy relevance of eco-affective measures under real-world conditions [11]. Comparative analyses across sites with similar environmental profiles but distinct affective patterns can clarify how eco-affective variables explain divergence in resilience trajectories. Over the medium term, efforts should focus on context-sensitive standardization. Harmonized measurement protocols, combined with contextual calibration, can support meaningful comparison while preserving local specificity [7,14].

A second priority is integration with environmental and exposure datasets. Linking eco-affective indicators to longitudinal environmental data enhances interpretability and supports dynamic system analysis [1,4,16,17]. In the long term, eco-affective integration has the potential to reshape resilience itself. Rather than focusing solely on robustness or recovery, resilience can be reframed as the capacity of coupled human-environment systems to sustain function, legitimacy, and collective engagement over time [6].

## Conclusion

Resilience science has made substantial progress in translating a once abstract concept into operational tools for policy, planning, and governance [6]. Yet current approaches remain structurally incomplete. By prioritizing material and institutional dimensions, resilience quantification often overlooks the human experience through which environmental change becomes socially consequential. This omission limits both explanatory power and anticipatory capacity. Eco-affective state variables provide a pathway to address this limitation. As population-level affective signals, they capture early indicators of adaptive strain and system vulnerability, complementing existing metrics rather than replacing them [1,11].

This perspective extends resilience science by incorporating how environmental change is experienced and sustained at the population level. As environmental pressures intensify, resilience cannot be meaningfully quantified if human experience remains outside system boundaries. Integrating eco-affective variables represents a necessary step toward more complete and actionable models of resilience, capable not only of describing system performance, but of anticipating whether systems can endure.
